# Relationship between psychological capital and depression in Chinese physicians: The mediating role of organizational commitment and coping style

**DOI:** 10.3389/fpsyg.2022.904447

**Published:** 2022-11-17

**Authors:** Li Sun, Yuan Zhang, Jingyun He, Kexin Qiao, Ce Wang, Shuai Zhao, Jinxin Zhao, Xiaohui Qiu, Xiuxian Yang, Jiawei Zhou, Tianyi Bu, Xiaomeng Hu, Zhengxue Qiao, Depin Cao, Yanjie Yang

**Affiliations:** ^1^Department of Medical Education Management, School of Health Management of Harbin Medical University, Harbin, China; ^2^Harbin Medical University Cancer Hospital, Harbin, Heilongjiang, China; ^3^Psychology and Health Management Center, Harbin Medical University, Harbin, Heilongjiang, China

**Keywords:** psychological capital, organizational commitment, positive coping styles, depression, serial multiple mediation model

## Abstract

**Background:**

It is well known that the medical profession is a high-risk practice, with intense work and complex situations. The physicians are prone to suffer from depression due to occupational stress under high workloads for long periods of time. Depression not only impairs physicians’ mental health, but also affects the quality of health services, so it is important to explore the factors and mechanisms affecting depression among physicians.

**Methods:**

In this cross-sectional study, 1,139 physicians from Heilongjiang Province were surveyed by cluster sampling procedures. The questionnaires included Psychological Capital Questionnaire, Chinese Employee Organizational Commitment Questionnaire, Trait Coping Style Questionnaire and Self-rating Depression Scale. Pearson correlation analysis and Bootstrap were used for statistical analysis.

**Results:**

This study found that 41.6% of physicians suffering from depression. Among them, 17.0% of physicians showed moderate depression, and 2.6% of physicians exhibited severe depression. The serial-multiple mediation of organizational commitment and positive coping styles in the relationship between psychological capital and depression was significant.

**Conclusion:**

The results showed that the psychological capital was sequentially associated with increased organizational commitment, and then increased positive coping styles, which resulted in reduced depression among physicians.

## Introduction

Today, as the reform of China’s medical system is accelerating, both hospitals and physicians are facing increasing pressure for survival and development. In addition, due to frequent late nights and overtime work and the complexity of patients’ conditions, physicians are highly stressed for long periods of time. Furthermore, the lack of understanding from patients and their families, as well as frequent workplace violence, leads to tension in the physician-patient relationship. For a series of reasons, physicians work under great pressure and are prone to suffer from depression.

Studies found that physicians have increased incidence of depression. A British study showed that 29% of physicians in the intensive care unit of a hospital were depressed, and 3% of them had suicidal ideations (Coomber et al., 2002). According to a systematic review and meta-analysis of depression and depressive symptoms among residents, the prevalence of depression or depressive symptoms of physicians was 28.8% (Douglas et al., 2015). Reuben (Reuben, 1985) concluded that the prevalence of depression among physicians decreased with increasing work experience and income; however, depression levels are still higher than in the general population (Caplan, 1994). Depression symptoms among physicians not only affect physicians’ mental health, but also increase medical errors and reduce the quality of patient care (Landrigan et al., 2004; Petra et al., 2019). Therefore, it is crucial to explore the protective factors against depression in order to reduce the occurrence of depression.

Psychological capital has been reported to be a protective factor for depressive symptoms (Hao et al., 2015). Psychological capital is a positive psychological state that individuals display during their growth and development (Luthans et al., 2007b). It consists of four main dimensions: self-efficacy (self-confidence), hope, optimism and resiliency. Self-efficacy refers to a person’s belief (or confidence) in his or her ability to mobilize the motivation, cognitive resources, and course of action required to successfully execute a particular task in a given context; hope refers to a positive, motivation-related state, based on a sense of success resulting from the interaction between goal-directed initiative and pathways to meet goals; optimism is a person’s ability to attribute positive events to positive explanatory styles in which a person attributes positive events to personal, permanent, and generally pervasive causes; resiliency refers to a person’s ability to rebound quickly from adversity, conflict, and failure, or even positive events, progress, and increased responsibility (Luthans et al., 2007b; Luthans and Youssef, 2007). Psychological capital has been shown to be an important influence on positive outcomes in many studies, and it can promote well-being (Luthans and Youssef-Morgan, 2015). A study of 1,300 physicians in Liaoning, China, showed a significant positive correlation between psychological capital and professional effectiveness (Wang et al., 2012). It has also been shown that psychological capital is positively related to both performance (Luthans et al., 2007a) and job satisfaction (Larson and Luthans, 2006). To explain how psychological capital affects outcome variables, a dynamic effects model proposed by Wang et al. suggests that psychological capital can influence outcome variables directly and indirectly (Wang and Zhu, 2007). Therefore, we believe that psychological capital not only can directly affect depressive symptoms, but also indirectly affect depression through other influencing factors.

Some studies claim that psychological capital is a positive resource for improving organizational commitment (Laschinger and Grau, 2012). The concept of organizational commitment was developed by American academic Becker (1960). Organizational commitment is considered to be a psychological link between employees and the organization that reduces the possibility of voluntary turnover (Allen and Meyer, 1996). Psychological capital is positively correlated with organizational commitment, and employees with job commitment can reduce the turnover intention of their company (Humayra and Mahendra, 2019). Also it has been shown that psychological capital influences burnout through the mediating role of organizational commitment (Peng et al., 2013; Zhou et al., 2018). In addition, a large number of studies have proved that psychological capital is a predictor of organizational commitment (Luthans and Jensen, 2005). This can also be supported by the antecedent outcome model of organizational commitment proposed by Steers in 1977, which states that the antecedent variables of organizational commitment include personal characteristics and job characteristics, and the outcome variables of organizational commitment mainly include intention to stay, attendance, etc. (Steers, 1977). As a personal characteristic, the psychological capital will have an impact on organizational commitment, while the coping styles will also be reflected in the outcome variables such as intention to stay in the job in a behavioral way, which laterally indicates the impact of organizational commitment on coping styles. Studies found that problem-solving coping styles play a partially mediating role in the relationship between affective commitment and positive emotion, as well as in the relationship between sustained commitment and positive emotion (Marchand and Vandenberghe, 2013). Thus, we hypothesized that organizational commitment would play a mediating role in the relationship between psychological capital and positive coping styles.

Coping styles refer to the cognitive strategies and behaviors that individuals adopt to meet the requirements when they face events beyond their capacity (Folkman et al., 1986). It plays an important role in mental health as a mediating mechanism between stress and health (Spector, 1988). Coping styles have been reported to partially mediate the relationship between psychological capital and psychological distress (Zhou et al., 2017). Psychological capital affects individual coping style or coping mechanism (Pan and Zhou, 2009), and coping styles affect psychological distress. The effect of coping styles on depression has been demonstrated in many studies, and positive coping styles are considered to be a protective factor against depression (Roohafza et al., 2014). Therefore, we hypothesized that coping styles play a mediating role in the relationship between psychological capital and depression.

The aim of this study was to explore the role of organizational commitment and positive coping styles in the relationship between psychological capital and depression. We hypothesize that psychological capital affect depression through the chain mediating effect of organizational commitment and positive coping styles.

## Materials and methods

### Participants

The survey was conducted in Daqing City, Heilongjiang Province, China. There are 5 comprehensive hospitals of tertiary grade A (>500 beds) in Daqing, which were labeled as 1–5, and two of them were randomly selected for cluster sampling procedures. Among 1,286 physicians of two hospitals, a total of 1,139 subjects were recruited from different clinical departments. A self-administered questionnaire was provided to each physician by trained investigators. After excluding participants with invalid data, 1,081 valid questionnaires were obtained, with a valid recovery rate of 94.90%. Among 1,081 physicians, 447 were male, accounting for 41.4%; and 634 were female, accounting for 58.6%, with an average age of (38.95 ± 7.60) years old. The medical specialties of Physicians include Surgery, Respiratory, Cardiology, ophthalmology, Neurology, Pediatrics, etc. The study was approved by the Ethics Committee of the Harbin Medical University and all participants gave their written consent.

### Measurement of psychological capital

The Psychological Capital Questionnaire (PCQ), developed by Luthans et al. (2007b), is a 24-item self-report scale that includes four dimensions: self-efficacy, optimism, resiliency, and hope. The items are rated from 1 (strongly disagree) to 6 (strongly agree), and the Cronbach’s alpha coefficient for the PCQ was 0.925.

### Measurement of organizational commitment

The study used 25-item Chinese Employee Organizational Commitment Questionnaire (OCQ for Chinese) developed by Chinese scholars to measure the organizational commitment of physicians. The questionnaire includes five dimensions; affective commitment, normative commitment, ideal commitment, economic commitment and opportunity commitment. Each dimension consists of five items, and each item is scored on a five-point scale from 1 (strongly disagree) to 5 (strongly agree). The scale score was the sum of all items and total scores ranged from 25 to 125. The Cronbach’s was 0.870 in this study.

### Measurement of coping styles

The Trait Coping Style Questionnaire (TCSQ) was compiled by Jiang Qianjin. The TCSQ consists of 12 items, including two dimensions, and it is scored on a five-point scale from 1 (definitely not) to 5 (definitely yes). The two dimensions included positive coping styles and negative coping styles. The Cronbach’s α was 0.803 in this study.

### Measurement of depression

Self-rating Depression Scale was used to measure the severity of depressive symptoms among the physicians. It consists of 20 items. Each item is graded from 1 (never) to 4 (all the time). The total score was calculated as the sum of 20 items. The Cronbach’s α was 0.871 in this study.

### Statistical analysis

The SPSS package (version 20.0 for Windows) was used to analyze the data. Pearson correlation analysis to explore the relationship between psychological capital, organizational commitment, coping styles and depression; In the complementary mediation analysis, model 6 of PROCESS macro was used to explore the relationship between independent variable (psychological capital) and dependent variable (depression), which may be affected by mediator variable1 (organizational commitment) and mediator variable 2 (positive coping styles).

## Results

### Depression

As shown in Table 1, the total percentage of depression was 41.6%, and among them，the percentage of mild, moderate, and severe depression were 22.0, 17.0 and 2.6%, respectively.

**Table 1:** The distribution of different levels of depression.

Degree	Number	%	Mean score
Normal	631	58.4	38.42 ± 6.58
Mild depression	238	22.0	53.99 ± 2.78
Moderate depression	184	17.0	62.97 ± 2.20
Severe depression	28	2.6	75.22 ± 5.17
Total	1,081	100.0	46.98 ± 12.10

### Association between psychological capital, organizational commitment, coping styles and depression.

As shown in Table 2, psychological capital was positively related to organizational commitment (*r* = 0.366, *p* < 0.01) and positive coping styles (*r* = 0.556, *p* < 0.01), and psychological capital was negatively related to depression (*r* = −0.556, *p* < 0.01). Organizational commitment was positively related to positive coping styles (*r* = 0.315, *p* < 0.01), and organizational commitment was negatively related to depression (*r* = −0.200, *p* < 0.01). Positive coping styles were negatively associated with depression (*r* = −0.443, *p* < 0.01).

**Table 2:** Correlations between psychological capital, organizational commitment, positive coping styles and depression.

Variables	M ± SD	Psychological capital	Organizational commitment	Positive coping styles	Depression
Psychological capital	104.63 ± 16.14	1			
Organizational commitment	79.44 ± 13.03	0.366**	1		
Positive coping styles	34.95 ± 7.13	0.556**	0.315**	1	
Depression	46.98 ± 12.10	−0.556**	−0.200**	−0.443**	1

**p < 0.01.

**Figure 1:**
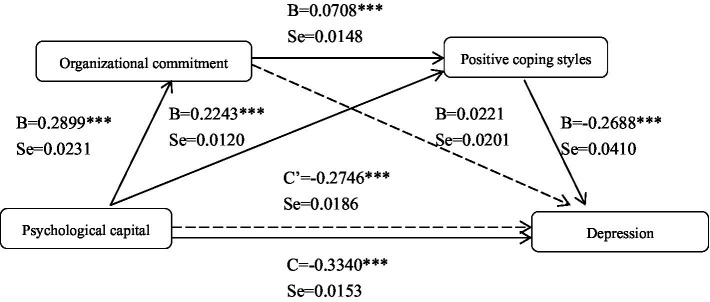
The mediating path of depression. B represents the direct effect coefficient of the antecedent variable on the outcome variable conditional on gender and age as covariates, c’ represents the direct effect coefficient of psychological capital on depression conditional on gender and age as covariates and organizational commitment and positive coping style being included in the mediating model, and c represents the full effect coefficient of psychological capital on depression.

The results of organizational commitment and positive coping styles played a chain mediating role between psychological capital and depression was shown in Figure 1 and Table 3.

**Table 3:** Mediating role analysis of depression of physicians.

Path	Effect value	Boot SE	%	*t*	*p*	Bootstrapping 95% BC Confidence interval (CI)	
						BootLL CI	BootUL CI
Direct effect	−0.2746	0.0186	82.22%	−14.8002	0.000	−0.3110	−0.2382
Indirect effect	−0.0594	0.0125	17.78%			−0.0842	−0.0348
X-M1-Y	0.0064	0.0070	1.92%			−0.0068	0.0207
X-M2-Y	−0.0603	0.0106	18.05%			−0.0815	−0.0400
X-M1-M2-Y	−0.0055	0.0017	1.65%			−0.0093	−0.0026

M1, Organizational commitment; M2, Positive coping styles.

In Figure 1, the total effect (*c* = −0.3340, SE = 0.0153, *p* < 0.001) of psychological capital on depression was found to be significant. In addition, psychological capital had a positive direct effect on organizational commitment (*B* = 0.2899, SE = 0.0231, *p* < 0.001) and positive coping styles (*B* = 0.2243, SE = 0.0120, *p* < 0.001). The direct effect of organizational commitment as the first mediating variable on the second mediating variable of positive coping styles (*B* = 0.0708, SE = 0.0148, *p* < 0.001) was also found to be significant. The direct effect of mediating variables on depression showed that the effect of positive coping styles (*B* = −0.2688, SE = 0.0410, *p* < 0.001) was significant. When psychological capital and the two mediating variables were simultaneously entered into the model, the direct effect of psychological capital on depression was also found to be significant (*c*’ = −0.2746, SE = 0.0186, *p* < 0.01). Overall, these results revealed that serial-multiple mediation was established.

As shown in Table 3, the mediating effect of positive coping styles in the relationship between psychological capital and depression was significant (95% CI: −0.0815, −0.0400). The serial-multiple of organizational commitment and positive coping styles in the relationship between psychological capital and depression was significant (95% CI: −0.0093, −0.0026). The direct effect of psychological capital on depression was significant with a 95% confidence interval (−0.3110, −0.2382).

## Discussion

To the best of our knowledge, this is the first study examining the role of organizational commitment and positive coping styles in the relationship between psychological capital and depression in Chinese physicians. The results found that 41.6% of physicians suffering from depression. In a survey of 1,488 physicians in Liaoning Province, 65.3% of physicians reported symptoms of depression (Wang et al., 2010). There is also a domestic study of primary care physicians in Shanghai found that the prevalence of depressive symptoms is 31.8% (Shen et al., 2012). The prevalence of depression among emergency department residents in the United States was 12.1% (Katz et al., 2014). Moreover, A cross-sectional study of primary care physicians in Shanghai showed that the score of depressive symptom was 46.5 ± 11.8, which is similar to the results of this study (Shen et al., 2012). One study showed a depression score of 14.20 ± 9.20 for intensive care physician by using the CES-D 20 scale (the total score is 60; Nathalie et al., 2012). Therefore, the prevalence of depressive symptoms among Chinese physicians is still at a high level.

First, psychological capital is significantly correlated with depression, which is consistent with the results of previous research (Liu et al., 2012), confirming that there is a negative correlation between psychological capital and depressive symptoms. In addition, psychological capital was positively correlated with positive coping styles and then negatively correlated with depression, which confirmed the partial mediating role of positive coping styles in the relationship between psychological capital and depression. In other words, this study found direct and indirect effects of psychological capital on depression mediated *via* positive coping styles. This is consistent with previous studies (Zhou et al., 2017). When facing difficulties, physicians with higher psychological capital have higher energy to cope with them on one hand, and tend to regard problems as challenges on the other hand. Therefore, they are more likely to adopt positive coping styles. Taking a positive coping style helps to solve the problem and then get a sense of achievement, thus reduce the occurrence of depression.

Psychological capital was also found to influence depression through a chain mediating effect of organizational commitment and positive coping styles. Psychological capital was first associated with high levels of organizational commitment and then positively associated with positive coping styles. Positive coping styles is negative correlated with depression. Employees with high levels of psychological capital are more likely to identify with their team, to love their work, and to actively contribute to the organization (Larson and Luthans, 2006; Zhong, 2007). As a result, such employees have a higher level of organizational commitment. A high level of commitment to the organization indicates that they are satisfied with the people, things and treatment level in the organization, are highly consistent with the goals of the organization, with great enthusiasm and energy for work, so they will adopt positive coping styles. Physicians who are more likely to adopt positive coping styles will face problems at work with a positive attitude and find positive solutions. Therefore, they are less likely to suffer from depression.

As physicians play a critical role in medical and health services, it is essential to take effective measures to reduce depression of physicians. First, more training opportunities and health education should be provided to improve the knowledge and experience of physicians and raise their sense of psychological capital; Second, create a relaxed, pleasant, sincere and efficient working atmosphere to improve team cohesion and enhance the organization commitment of physicians; Third, psychological education lectures could be carried out to encourage physicians to cope with stress and to solve problems with a positive coping styles; Finally, effective psychological intervention should be taken to reduce the depression of physicians.

This study has some limitations. First, the present study was a cross-sectional study and the results should be interpreted cautiously. Second, all questionnaires were self-reported and there may be biases in recollection of data. Finally, the physicians in this study were from Heilongjiang province, the results of present study could not be generalized to all physicians in China, multi-centered research should be conducted in the future.

## Data availability statement

The raw data supporting the conclusions of this article will be made available by the authors, without undue reservation.

## Ethics statement

The studies involving human participants were reviewed and approved by Ethics Committee of the Harbin Medical University. The patients/participants provided their written informed consent to participate in this study.

## Author contributions

ZQ, DC, and YY designed the study and critically reviewed the article. JH, JXZ, SZ, CW, KQ, and TB participated in the investigation. XQ, XY, JWZ, and XH analyzed the data. LS and YZ wrote the article. All authors contributed to the article and approved the submitted version.

## Funding

This research was supported by the Natural Science Foundation of Heilongjiang Province, China (LH2021H005) to ZQ and National Natural Science Foundation of China (81773536) to YY.

## Conflict of interest

The authors declare that the research was conducted in the absence of any commercial or financial relationships that could be construed as a potential conflict of interest.

## Publisher’s note

All claims expressed in this article are solely those of the authors and do not necessarily represent those of their affiliated organizations, or those of the publisher, the editors and the reviewers. Any product that may be evaluated in this article, or claim that may be made by its manufacturer, is not guaranteed or endorsed by the publisher.
